# The Effect of Dietary Types on Gut Microbiota Composition and Development of Non-Communicable Diseases: A Narrative Review

**DOI:** 10.3390/nu16183134

**Published:** 2024-09-17

**Authors:** Martin Soldán, Ľubica Argalášová, Lucia Hadvinová, Bonzel Galileo, Jana Babjaková

**Affiliations:** Institute of Hygiene, Faculty of Medicine, Comenius University in Bratislava, Špitálska 24, 813 72 Bratislava, Slovakia; soldan9@uniba.sk (M.S.); lucia.hadvinova@fmed.uniba.sk (L.H.); bonzel1@uniba.sk (B.G.); jana.babjakova@fmed.uniba.sk (J.B.)

**Keywords:** gut microbiota, dietary types, non-communicable diseases, narrative review

## Abstract

Introduction: The importance of diet in shaping the gut microbiota is well established and may help improve an individual’s overall health. Many other factors, such as genetics, age, exercise, antibiotic therapy, or tobacco use, also play a role in influencing gut microbiota. Aim: This narrative review summarizes how three distinct dietary types (plant-based, Mediterranean, and Western) affect the composition of gut microbiota and the development of non-communicable diseases (NCDs). Methods: A comprehensive literature search was conducted using the PubMed, Web of Science, and Scopus databases, focusing on the keywords “dietary pattern”, “gut microbiota” and “dysbiosis”. Results: Both plant-based and Mediterranean diets have been shown to promote the production of beneficial bacterial metabolites, such as short-chain fatty acids (SCFAs), while simultaneously lowering concentrations of trimethylamine-N-oxide (TMAO), a molecule associated with negative health outcomes. Additionally, they have a positive impact on microbial diversity and therefore are generally considered healthy dietary types. On the other hand, the Western diet is a typical example of an unhealthy nutritional approach leading to an overgrowth of pathogenic bacteria, where TMAO levels rise and SCFA production drops due to gut dysbiosis. Conclusion: The current scientific literature consistently highlights the superiority of plant-based and Mediterranean dietary types over the Western diet in promoting gut health and preventing NCDs. Understanding the influence of diet on gut microbiota modulation may pave the way for novel therapeutic strategies.

## 1. Introduction

The gut microbiota is a dynamic complex of microorganisms located in the gastrointestinal tract of humans [1]. It includes *Bacteria*, *Archaea*, viruses, and protists, most of which engage in a symbiotic relationship with the host. However, certain conditions can turn commensal bacteria into parasites [2,3]. As a pivotal part of the human body, gut microbiota plays a significant role in nutrition and physiology [4]. It is often suggested to be a superorganism [5]. So far, over 2000 species have been identified, with 12 distinct phyla. The majority of isolated species, 93.5% were *Proteobacteria*, *Actinobacteria*, *Firmicutes*, and *Bacteroidetes* [6]. *Firmicutes* make up 65% of the total gut bacteria, *Bacteroidetes* 23%, *Actinobacteria* 5%, and the fourth most represented phyla are *Proteobacteria* [7].

Diet is a fundamental factor for maintaining healthy status and preventing non-communicable diseases (NCDs), such as cancer, cardiovascular diseases, and cognitive or metabolic impairments. NCDs represent the majority of cases of mortality all around the world. The gut microbiota is essential in the absorption and extraction of nutrients. It is thus well known that diet has a great influence on its formation, which participates in the pathogenesis of NCDs [8,9,10]. The gut microbiota closely interacts with the immune system and metabolism. Changes in a diet not only affect the gut bacteria but also have an impact on bodily functions. For instance, the gut microbiota plays a role in responding to infections or regulating the appetite, as some microbial products influence the hypothalamic satiety pathway. A shift in microbial equilibrium caused by dietary changes leads to dysbiosis, which is related to many disorders. Therefore, a balanced diet with antioxidant and anti-inflammatory components represents a valuable way to prevent multiple NCDs [5,7].

Both macronutrients, such as fiber, protein, and fatty acids, and micronutrients, including minerals and vitamins, significantly impact gut microbiota diversity and composition. Both long-term and short-term dietary interventions have proven to be effective. For instance, a diet high in fiber may lengthen the lifespan by reducing systemic inflammation, a result of fiber fermentation by-products. Rural communities tend to have more diverse gut microbiota, which is linked to their high fiber consumption and enhanced Bacterial capacity for fiber fermentation. Similar health benefits have been seen in individuals following a diet rich in whole grains and total carbohydrates. In comparison, diets rich in fats and low in fiber, such as the Western diet, are associated with increased inflammation levels. The Westernization of a diet also reduces bacterial diversity, and some bacteria may even be absent compared to rural populations [5,11,12].

Nutritional content differs among various dietary patterns. However, focusing on a single nutrient is not ideal for determining the impact of a diet on an individual. Consequently, studying dietary patterns is more appropriate, as they provide better information about various interactions between nutrients, both synergistic and antagonistic. Synergistic food interactions between food components and dietary patterns have a profound effect on overall health. Current evidence suggests that despite the fact that some nutrients might have an impact on the prevention of chronic disorders, no isolated component is protective. This is due to the complexity of food acting on various biochemical pathways, which is especially relevant for chronic diseases such as cancer, cardiovascular diseases, obesity, and type 2 diabetes. In addition, paying attention only to nutrients might also have contradictory outcomes. For example, a diet rich in saturated fats is linked to a higher risk of cardiovascular diseases. However, diets high in carbohydrates and low in fats can lead to an overconsumption of added sugars and refined carbohydrates, further multiplying the cardiovascular risk [13,14].

The aim of this narrative review is to assess the relationship between three dietary types (plant-based diet, Mediterranean diet, and Western diet) and the bacterial composition of the gut. Furthermore, we evaluate the impact of gut dysbiosis on the development of NCDs.

## 2. Methods

This review employs a narrative approach to analyze publications in the field and to highlight the importance of different dietary patterns and their effects on gut microbiota composition, as well as the development of diseases related to gut microbiota. This narrative review was conducted using the PubMed, Web of Science, and Scopus databases for a literature search, with an emphasis on the keywords “dietary pattern”, “gut microbiota” and “dysbiosis”. The inclusion criteria were limited to English-language papers related to gut microbiota and the development of chronic diseases. The emphasis was placed on empirical studies as well as on systematic reviews that systematize state-of-the-art knowledge. Abstracts and full texts were independently reviewed by two authors, ensuring that the articles meet the selection criteria for this topic. All duplicates were removed, resulting in a final selection of 120 articles, written in English between 2005 and 2024, for inclusion in the narrative review.

## 3. Gut Microbiota Composition in the Gastrointestinal Tract

The distribution, quantity, and type of microorganisms vary throughout the gastrointestinal tract. While relatively few species inhabit the stomach and small intestine, the large intestine is home to a dense and diverse microbial community [15]. This is due to different physiology, pH, accessibility of substrates, oxygen partial pressure, flow rate of digestion, and host secretions [16]. At least 700 different bacterial species are present in the human mouth cavity [17]. More than 94% comprises six phyla, *Bacteroidetes*, *Firmicutes*, *Fusobacteria*, *Actinobacteria*, *Proteobacteria*, and *Spirochaetes.* The distribution of bacteria is modified by saliva and soft- and hard-tissue surfaces. More essentially, the hard surface of the tooth enables the formation of biofilms, which establish a steady environment for bacterial growth [18]. In the esophagus, the most abundant genus is *Streptococcus* [19]. It is suggested that healthy stomach microbiota is mostly formed by *Prevotella*, *Veillonella*, *Streptococcus*, *Haemophilus*, and *Rothia.* In addition, *Bacteroidetes*, *Firmicutes*, *Fusobacteria*, *Actinobacteria*, and *Proteobacteria* were also found [20]. People who tested positive for *Helicobacter pylori* had this bacterium as the most represented. However, the presence of *Helicobacter pylori* does not affect the microbial diversity of the stomach [21].

The small intestine is divided into three sections: duodenum, jejunum, and ileum. The main function that the small intestine serves is the absorption of nutrients [22]. The microbiota is less diverse and numerous than in the colon, with a biological mass of 10^3^–10^7^ microbial cells per gram of intestinal tissue, mainly because of lower pH, pancreatic peptides, bile acids, and quicker transit time. It is assumed that the most abundant phyla are *Proteobacteria* and *Firmicutes*, which can withstand these factors [23]. Other frequently occurring bacteria are *Veillonella* and *Streptococcus* [22]. The large intestine comprises multiple parts, namely, the caecum, ascending, transverse, descending, sigmoid colon, and rectum. Anaerobic conditions of the large intestine provide a good environment for anaerobic microorganisms to grow [24]. The most represented bacterial phyla are *Bacteroidetes* and *Firmicutes*, which together make up 90% of the total bacteria population [25]. During life, the ratio between *Bacteroidetes* and *Firmicutes* varies, which can implicate the health of an individual. In terms of density, dominant bacterial genera, *Bacteroides*, *Bifidobacterium*, *Eubacterium*, *Clostridium*, *Propionibacterium*, *Peptostreptococcus*, and *Ruminococcus,* account for 10^9^ of bacterial cells/gram of colon tissue [26]. There are also pathogenic bacteria present in the large intestine, namely, *Escherichia coli*, *Vibrio cholerae*, *Bacteroides fragilis*, *Salmonella enterica* and *Campylobacter jejuni,* and they form around 0.1% of the total bacterial population [27].

## 4. Function of Gut Microbiota

The gut microbiota plays a critical role in the fermentation of dietary fiber (oligosaccharides, polysaccharides, pectin, lignin, and resistant starches). By doing so, it generates short-chain fatty acids (SCFAs), namely, acetate, butyrate, and propionate. The main bacterial phyla involved in SCFA production are *Firmicutes* and *Bacteroidetes* [28,29]. SCFAs are absorbed in the gastrointestinal tract (GIT) and represent a valuable source of energy for host cells [30]. SCFAs also have a variety of different effects, for example, regulation of gene expression, apoptosis, chemotaxis, activation of gluconeogenesis, and possibly regulation of appetite or maintaining the integrity of the GIT epithelium [31,32,33]. Acetate was reported to be involved in the production of IgA by intestinal B-cells and IgA plays a crucial role in the toleration of gut bacteria and fight against pathogenic bacteria [34].

By metabolizing lecithin, choline, and L-carnitine by gut bacteria, a trimethylamine (TMA) is formed. TMA is then oxidated in the liver, and TMA changes into trimethylamine-N-oxide (TMAO), a molecule with proatherogenic effects [35]. TMA is produced mainly by *Proteobacteria*, *Actinobacteria*, and *Firmicutes* phyla [36]. Additionally, gut bacteria synthesize essential vitamins, including vitamin K and several B-group vitamins, such as folate, which is crucial for DNA synthesis and repair. Folate is synthesized mostly by *Bifidobacteria* [37,38]. Furthermore, gut bacteria can break down primary bile acids, transforming them into secondary bile acids, which can be reabsorbed [39]. The gut microbiota is also associated with the activation of polyphenols. After the activation, polyphenols are absorbed in the portal system [40].

Research has revealed a connection between the gut microbiota and the brain. The gut–brain axis is a bidirectional communication network that links the central, autonomic, and enteric nervous system with the hypothalamic pituitary adrenal system. This axis is believed to influence multiple mechanisms, including satiety and digestive functions, as well as behavior and mood [41,42]. There are multiple pathways between the brain and the gut, neural, endocrine, and immunological. The vagus nerve and ENS mediate the neural pathway. The ENS comprises the Meissner’s plexus found in the intestinal submucosa and the Auerbach’s plexus in the muscular layer of the intestine. The ENS is not only interconnected by many neural fibers but is also closely linked with gut-associated lymphoid tissue (GALT). Endocrine pathways involve the production of various molecules by gut bacteria, such as serotonin, acetylcholine, dopamine, or gamma-aminobutyric acid (GABA). In terms of the immune system’s role in the gut–brain axis, pro-inflammatory cytokines have a toxic effect on the central nervous system (CNS). The synthesis of the pro-inflammatory cytokines can be induced by lipopolysaccharide (LPS), a structural part of Gram-negative bacteria [43].

## 5. Factors Influencing the Gut Microbiota

The gut microbiota is at early stages of life influenced by the mother’s microbiota, but various factors shape it later in life, for instance, infections, the immune system, diet, and the use of medication. Genetics play an essential role in the formation of gut microbiota and several bacterial species are heritable, such as *Actinobacteria* and *Firmicutes*. This can be demonstrated in monozygotic and dizygotic twins. Both types of twins, when sharing the same environment, show differences, but the difference between the gut microbiota of monozygotic twins is smaller [44]. Age is another important factor in gut microbiota composition. In infants, the gut microbiota lacks diversity when compared to adults. There is a large influx of bacteria until about 3 years of age when the gut microbiota starts to develop adult characteristics as the diet transitions from milk to solid foods [45,46]. In adult humans, the structure and function of gut microbiota stay stable, but events such as diseases and antibiotic treatments may cause an alteration by changing bacterial composition and transferring genes of antibiotic resistance [1,47,48]. In the elderly population (over 65 years old), bacterial diversity decreases and there is a shift towards facultative anaerobic bacteria at the expense of beneficial bacteria [49].

Lifestyle factors, including alcohol consumption, smoking, and physical activity, further modulate gut microbiota. Alcohol promotes dysbiosis through two mechanisms: a change in gut microbiota composition and a reduction in nutrient absorption. Smoking as a risk factor acts in many ways, for instance, by modifying pH and oxygen levels, and through the production of acid in the gastrointestinal tract. Tobacco is also an immunosuppressant. Exercise can enhance the diversity of gut microbiota and is even suggested as a remedy for dysbiosis-related chronic illnesses. However, it is difficult to prove the beneficial effects of exercise on the gut microbiota of athletes since most of them have a distinct diet [50,51,52,53,54,55,56].

Diet and geography are important factors involved in the diversity of gut microbiota [57]. When comparing industrialized and non-industrialized countries, there is a greater *Bacteroidetes*-to-*Firmicutes* ratio [55]. Another division is by using enterotypes. Three types can be distinguished: enterotype 1, characterized by an abundance of *Bacteroides*; enterotype 2, rich in *Prevotella;* and enterotype 3, where *Firmicutes* are large in number, especially *Ruminococcus* [58]. Higher altitudes suit anaerobic bacteria, and during cold environmental stress, there is a change in the *Bacteroidetes*-to-*Firmicutes* ratio in favor of Firmicutes [59,60].

## 6. Diseases Related to Gut Microbiota

Dysbiosis is a term that indicates the change and imbalance in the gut microbiota’s composition, which can lead to various illnesses [61]. As regards gastrointestinal diseases, for instance, irritable bowel syndrome (IBS) is characterized by altered gut–brain axis signaling, a change in the motility of GIT, increased visceral sensitivity, and impaired gut barrier function [62]. In terms of gut bacteria composition of IBS patients, the *Bifidobacterium* and *Faecalibacterium* populations were reduced. On the other hand, *Proteobacteria*, *Bacteroides*, and *Lactobacillacae* showed higher numbers in contrast to the control group [63]. Inflammatory bowel disease (IBD) consists of Crohn’s disease and ulcerative colitis [64]. Numerous changes in the gut microbiota of IBD patients have been reported, namely, a higher abundance of *Candida tropicalis*, *Escherichia coli*, and a decrease in *Firmicutes*, *Bacteroidetes*, or *Faecalibacterium prausnitzii* in comparison with the control group [55,65,66]. The pathogenesis of colorectal cancer (CRC) is characterized by a greater number of pathogenic bacteria. For example, *Bacteroides fragilis* and *Escherichia coli* promote a chronic inflammation of intestinal tissue, which can lead to the development of CRC [67].

It was believed that the *Firmicutes*/*Bacteroidetes* ratio changes during the pathogenesis of obesity; however, this has been recently proven untrue, as there is no microbiological connection to human obesity [68]. Dysbiosis of the gut microbiota is a factor connected to the pathogenesis of diabetes mellitus (DM), both type 1 and type 2 [69]. To be more specific, type 1 DM patients exhibit a higher abundance of *Ruminococcus* and *Bacteroides*, with a lower proportion of *Prevotella* and *Clostridium* as opposed to the control group. In type 2 DM, there is a drop in *Akkermansia* and *Bifidobacterium* amounts [70,71,72]. Furthermore, type 2 DM represents a good environment for the growth of opportunistic pathogens, for example, *Escherichia coli* or *Clostridium symbiosum* [73]. This growth is due to higher concentrations of pro-inflammatory cytokines. Some bacteria, like *Lactobacillus,* can inhibit the production of pro-inflammatory IL-1β. Similarly, *Akkermasia* can decrease concentrations of TNF-α, and *Bifidobacterium* species may even have positive effects on glucose tolerance [74]. On the other hand, *Escherichia coli* is enhanced in prediabetic patients, individuals with already altered glucose tolerance [75].

It is suggested that the gut–brain axis might be associated with neuroinflammation, a process that leads to the loss of neurons, which is typical for Alzheimer’s disease (AD) and Parkinson’s disease (PD). PD patients had a higher abundance of *Enterobacteriaceae* and reduced populations of *Prevotellaceae.* AD is influenced by pathogenic bacteria like *Mycobacterium tuberculosis*, *Staphylococcus aureus*, or *Salmonella* spp. [76,77]. The gut–brain axis also seems to be linked to the pathogenesis of depression and anxiety [42]. Autism spectrum disorder (ASD) is another neurological disability in which the dysbiosis of gut microbiota is suggested to be involved, but the evidence is conflicting [78].

In addition, gut bacteria are also affiliated with cardiovascular disorders. As already mentioned, SCFAs and TMAO are metabolites produced by gut bacteria. SCFAs seem to have beneficial antihypertensive effects [79]. Conversely, TMAO plays a role in atherosclerosis’s pathogenesis by increasing cholesterol absorption by macrophages. Another mechanism might be linked to a decrease in HDL cholesterol concentrations. Moreover, TMAO has been linked to the activation of platelets [36]. Another molecule synthesized by the gut microbiota is phenylacetylglutamine (PAG). PAG is connected to acute cardiovascular events, like stroke, heart attack, or sudden death [79].

## 7. The Effect of Diets on Gut Microbiota Composition

**Figure 1:**
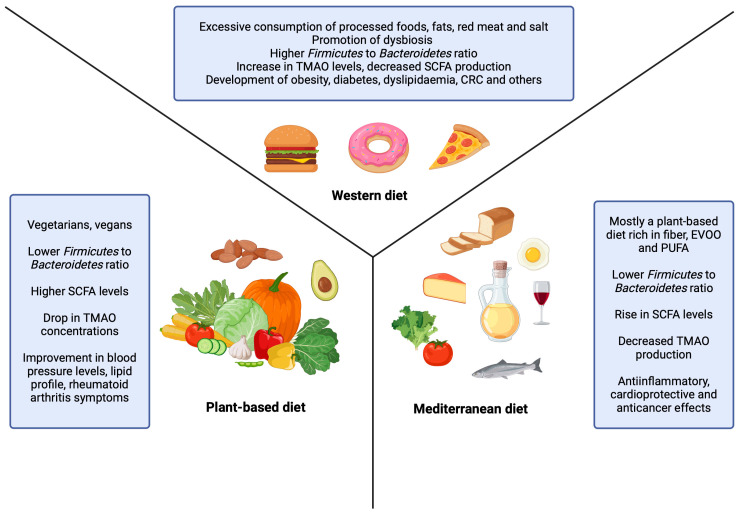
Characteristics of three distinct dietary types. Abbreviations: SCFAs—short-chain fatty acids, TMAO—trimethylamine-N-oxide, CRC—colorectal cancer, PUFAs—polyunsaturated fatty acids, EVOO—extra virgin olive oil. Created in BioRender. Soldán, M. (2024) BioRender.com/a05r617 (accessed on 8 September 2024).

Plant-based diets primarily consist of fruits, vegetables, seeds, nuts, legumes, whole grains, and herbs [80]. There are several types of plant-based diets, each with different restrictions on certain food groups. Flexitarians, for example, rarely consume meat, while pescatarians include fish and seafood as their only sources of animal protein. Ovolactovegetarians exclude meat products but consume dairy or eggs, whereas vegans adhere to a diet entirely composed of plant-based foods [81]. The Mediterranean diet is inspired by the traditional dietary patterns of countries bordering the Mediterranean Sea. It is predominantly a plant-based diet, with olive oil serving as a main source of fat. Animal products are consumed in moderation [82,83]. Other aspects of the Mediterranean diet are adequate intake of fish and dairy products with moderate consumption of red wine, which is served only during meals [84]. Conversely, the Western diet is often regarded as an unhealthy diet due to its excessive consumption of processed and refined foods, simple sugars, sweets, and animal fats, coupled with an inadequate intake of fruits, vegetables, nuts, and whole grains [7,85,86,87]. As shown in Figure 1, plant-based and Mediterranean diets lead to an increase in SCFA levels while simultaneously decreasing TMAO production. The *Firmicutes*/*Bacteroidetes* ratio is lower, and various health benefits are associated with these dietary patterns. On the contrary, the Western diet promotes the development of multiple chronic diseases and dysbiosis and causes an increase in TMAO levels together with a drop in SCFA production (Figure 1).

### 7.1. Plant-Based Diet

In 2023, there was a systematic review conducted by Sidhu et al. on the influence of plant-based diets on gut microbiota composition and the benefits of such diets in managing inflammatory and metabolic disorders. They included randomized control trials, non-randomized control trials, and pre–post interventions that explored the impact of a plant-based diet on gut microbiota. The systematic review analyzed 12 interventional studies, incorporating data from a total of 583 participants, aged 21 to 61 years, including both men and women. Participants varied in health status, comprising healthy individuals, those with obesity, rheumatoid arthritis, and individuals at cardiovascular risk. They adhered to a plant-based diet for periods ranging from 5 days to 13 months. A higher abundance of *Ruminococcaceae* and a decreased population of *Bacteroidaceae* were revealed in individuals following plant-based diets. Notably, differences were observed between vegan and vegetarian diets: vegans exhibited higher levels of *Coprococcus* and *Faecalibacterium*, while these bacterial populations were reduced in vegetarians. Additionally, improvements were noted in patients with rheumatoid arthritis and those with cardiovascular disorders, with optimized lipid profiles and blood pressure levels. These positive outcomes may be attributed to metabolic products like SCFAs or TMAO [88]. SCFAs, which are produced by the degradation of dietary fibers and carbohydrates, play an important role in the immune, metabolic, and neural systems [32]. The consumption of vegetables and fruits significantly influences SCFA levels, with a plant-based diet being shown to be superior in promoting SCFA production compared to an animal-based diet [88,89]. Conversely, an elevated TMAO level is associated with inflammation and metabolic diseases, mainly obesity and diabetes. Studies have shown that a plant-based diet can decrease TMAO levels compared to an animal-based diet [90]. In a study conducted by Djekic et al., comparing a vegetarian diet to a meat-dominant diet in patients with ischemic heart disease, a higher abundance of *Akkermansiaceae*, *Ruminococcaceae*, and *Lachnospiraceae* was found in subjects following the vegetarian diet. Consistent with previous findings, the vegetarian diet led to an increase in SCFA production and a decrease in TMAO levels, although this relationship was not statistically significant. However, a significant relationship between reduced plasma levels of L-carnitine (a TMAO precursor) and the vegetarian diet was demonstrated [91] (Table 1 and Table 2, Figure 1).

Trefflich et al. published a systematic review examining the relationship between gut microbiota composition and vegan or vegetarian diets compared to omnivorous diets. The review included 16 cross-sectional studies, primarily involving men and women aged 18 to 72. The total number of participants was 1229, consisting of 498 omnivores, 389 vegetarians, and 342 vegans who had adhered to a vegetarian or vegan diet for more than a month. The findings indicated that vegans had higher levels of *Bacteroidetes* compared to omnivores, while *Firmicutes* levels remained similar across the groups [110]. A study conducted by Kim et al. also addressed the *Firmicutes*/*Bacteroidetes* ratio. While obese individuals expressed a high *Firmicutes*/*Bacteroidetes* ratio, after adhering to a strict vegetarian diet, the ratio was reversed [103]. Additionally, according to Trefflich et al., vegetarians were reported to have increased levels of *Actinobacteria*. Among vegans, a decrease in *Proteobacteria* and an increase in *Verrucomicrobia* were found, both when compared to omnivores. Moreover, the *Clostridium* species quantity was substantially decreased in vegetarians compared to omnivores. However, this phenomenon was not observed in vegans, and the levels of *Faecalibacterium prausnitzii* were not fundamentally altered [110]. On the contrary, Kahleova et al. found that adherence to a vegan diet leads to a rise in *Faecalibacterium prausnitzii* populations in overweight individuals, along with a lower abundance of *Bacteroides fragilis* [92]. A plant-based diet also plays a role in gut microbiota composition during pregnancy. When comparing vegetarian and omnivorous pregnant women, *Roseburia* and *Lachnospiraceae* populations increased, while *Collinsella* and *Hholdemania* populations decreased [93]. Losno et al. investigated the composition of adult gut microbiota, comparing vegans and omnivores. Their results showed that *Bacteroidetes* were in higher numbers in vegans when compared to omnivores. Within *Bacteroidetes*, vegans exhibited an increase in *Prevotella*, but *Bacteroides* results were inconsistent. Furthermore, *Bifidobacteria* and *Enterobacteria* were less abundant in the vegan population than in omnivores. Interestingly, vegans also demonstrated a lower abundance of *Staphylococcus*, *Streptococcus*, and *Corynebacteria* compared to omnivores [111]. Raman et al. revealed different results. Individuals who followed a vegan diet and practiced meditation expressed a higher abundance of not only *Bifidobacteria*, but also *Lactobacillus*, *Streptococcus*, *Collinsella*, and *Ruminococcaceae* in contrast to the control group [94] (Table 1 and Table 2, Figure 1).

### 7.2. Mediterranean Diet

Kimble et al. assessed the effect of the Mediterranean diet on gut bacteria diversity, abundance, and metabolic products in a systematic review that evaluated 34 studies with a total of 4526 participants aged 22 to 95. While the majority of participants were healthy, individuals with various medical conditions were also included. Of the studies, 17 were observational, with 3 being prospective and 14 cross-sectional, while the other 17 were randomized control trials. Regarding bacterial diversity, a decrease in *Firmicutes* and an increase in *Bacteroidetes* populations in those who followed the Mediterranean diet were reported [82]. Similar results were described by Garcia-Mantrana, reporting that consuming a Mediterranean diet restricted in animal proteins leads to a lower *Firmicutes*/*Bacteroidetes* ratio [104]. In addition to that, following a hypocaloric Mediterranean diet reduces *Firmicutes* populations while increasing *Bacteroidetes* and *Proteobacteria* levels in obese individuals [95]. *Kimble* et al. also described a higher abundance of *Faecalibacterium prausnitzii*, which was also recognized in a study conducted by Meslier et al., where individuals following the Mediterranean diet displayed higher levels of *Faecalibacterium prausnitzii*, along with *Lachnospiraceae* and *Roseburia* [82,96] (Table 1 and Table 2, Figure 1).

Three core components of the Mediterranean diet are fiber, extra virgin olive oil (EVOO), and polyunsaturated fatty acids (PUFAs) [83]. It has been demonstrated that the consumption of EVOO leads to an expansion of *Lactobacillus* and reduced growth of pathogenic bacteria [112]. Similarly, PUFAs demonstrated anti-inflammatory and cardioprotective properties, with omega-3 (ω-3) PUFAs promoting the growth of *Bifidobacterium* while having an opposing effect on *Faecalibacterium* populations [7,113]. Dietary fiber is metabolized by gut bacteria, leading to the synthesis of SCFAs, which possess anticancer and cardioprotective properties [83]. Among SCFAs, a rise in propionate and butyrate is particularly notable in individuals following the Mediterranean diet, as observed in a study conducted by Seethaler et al. [106]. Similarly to a plant-based diet, TMAO levels are also decreased [108]. In addition, this diet has been shown to lower the risk of developing diabetes mellitus and other metabolic disorders [114]. So et al. conducted a systematic review and a meta-analysis where they analyzed the impact of dietary fiber on the composition of gut microbiota. While they did not identify any changes in alpha diversity, they did observe an increase in *Bifidobacterium* and *Lactobacillus* [115]. Other beneficial effects are attributed to a decreased oxidative state, inflammation, and positive impact on metabolic health represented by increased levels of *Eubacterium rectale* and *Clostridium leptum*, bacteria producing short-chain fatty acids, raising the levels of *Bacteroides*, *Bifidobacteria*, and *Faecalibacterium prausnitzii* species, and lowering the levels of *Blautia* species and *Firmicutes* [116]. However, a study by Rinott at al. noted reduced levels of *Bifidobacterium*, along with a higher *Prevotella* abundance after following the Mediterranean and Green Mediterranean diets [97]. Conversely, in previous observations, Diamanti et al. reported a drop in *Lactobacillaceae* and *Prevotella copri* populations in rheumatoid arthritis patients who showed greater adherence to the Mediterranean diet [98]. Additionally, Zhu et al. further explored the effect of a fiber-rich diet on gut microbiota. Their findings revealed improved microbial evenness and an increase in certain beneficial genera within the *Firmicutes* phylum. They also noted a positive effect of the fiber-rich diet on retrospective memory [117]. The Mediterranean diet is also rich in minerals, such as iron and zinc, both of which have an indisputable impact on overall health, including the activation of the immune system. However, iron can also promote the growth of pathogenic gut bacteria, leading to increased inflammation. The *Bifidobacteriacae* family can interact with iron by binding to it, therefore minimizing the negative effects of iron [7] (Table 1 and Table 2, Figure 1).

### 7.3. Western Diet

The health consequences of the Western diet are extensive, such as dyslipidemia, insulin resistance, systemic inflammation, overactivation of sympathetic and renin-angiotensin systems, as well as alterations in gut microbiota [87]. A major effect of the Western diet on the gut microbiota stems from the high consumption of processed and ultra-processed foods. Research has shown that factors such as acellular nutrients, artificial sweeteners, and emulsifiers can harm the gut microbiota and thus promote dysbiosis [118]. Another significant component of the Western diet is the excessive consumption of fats. Wolters et al. conducted a systematic review of the impact of fat on the composition of gut microbiota. They incorporated a total of 15 studies, out of which nine were cross-sectional observational studies and six randomized control trials. The number of participants varied widely, with cross-sectional studies ranging from 9 to 531 participants, and randomized controlled trials involving between 20 and 88 participants. Of these studies, 10 included both men and women, while two included only men and three only women with participants’ mean ages ranging from 8.1 to 63.3 years. Individuals followed a high-fat diet for 3 weeks up to a year. A reduced number of bacteria was observed in individuals following a diet high in monounsaturated fatty acids (MUFAs); however, *Prevotella*, *Enterobacteriaceae*, *Parabacteroides*, and *Turicibacter* populations grew. The ratio between *Firmicutes* and *Bacteroidetes* was also changed, with an increase in *Firmicutes* and decreased levels of *Bacteroidetes* [119]. Comparable results were presented by Nakayama et al., where children consuming a high-fat diet exhibited a higher *Firmicutes*/*Bacteroidetes* ratio [105]. Similarly, Wan et al. observed changes in gut microbiota following a high-fat diet, noting reduced levels of *Faecalibacterium*, while *Bacteroides* and *Alistipes* populations increased. In addition, a high-fat diet decreases the levels of specific SCFAs, including acetic acid, 2-hydroxybutyric acid, and 2-methylbutyric acid [99,107] (Table 1 and Table 2, Figure 1).

Other components of the Western diet might also affect the gut microbiota, for instance, refined carbohydrates, red and processed meat, and refined grains. Consumption of red meat leads to an increase in TMAO production. Moreover, the pathogenesis of CRC is strongly influenced by excessive consumption of red and processed meat. Gut dysbiosis may play a role in the CRC pathogenesis, namely, causing an increased volume of *Escherichia coli*, *Streptococcus bovis*, *Bacteroides fragilis*, and *Fusobacterium nucleatum*, which can create inflammation or alter the oncogenes and tumor-suppressing genes. However, Vanegas et al. found that the consumption of whole grains instead of refined grains leads to reduced numbers of pro-inflammatory *Enterobacteriaceae* [88,100,109,120]. Fried meat, as an unhealthy food preparation method, was shown in a study by Gao et al. to reduce gut microbiota diversity and cause alterations in bacteria abundances, including an increase in *Veillonella*, *Dorea*, and *Dialister*, while *Flavonifractor* and *Lachnospiraceae* levels were lower [101]. Additionally, added sugars may modulate the gut microbiota. Ramne et al. found that the consumption of sugar-sweetened beverages also adversely affects *Lachnobacterium* and leads to an increase in *Firmicutes* within the *Firmicutes*/*Bacteroidetes ratio* [102]. Furthermore, negative effects on insulin metabolism are associated with higher levels of *Clostridium bolteae* and *Blautia*, which are linked to diets high in short fatty acids (SFAs) [119]. As previously mentioned, systemic inflammation is a consequence of the Western dietary pattern. One of the main inflammation drivers is LPS. Increased gut permeability enhances the absorption of LPS, which may be attributed to a decrease in proteins that form the intestinal barrier, such as occludin and claudin [10] (Table 1 and Table 2, Figure 1).

## 8. Conclusions

The evidence reviewed in this article underscores the profound impact of diet on gut microbiota composition and, consequently, on health outcomes. Plant-based and Mediterranean diets, characterized by a high fiber intake and beneficial fats, are associated with enhanced production of SCFAs and reduced levels of TMAO, both of which are critical in reducing the risk of cardiovascular and metabolic diseases. These diets also promote greater microbial diversity, which is protective against a range of non-communicable diseases. In stark contrast, the Western diet, typified by high consumption of processed foods, fats, and sugars, is linked to gut dysbiosis, characterized by reduced microbial diversity, lower SCFA production, and elevated TMAO levels. This dysbiotic state significantly contributes to the development and progression of NCDs, including cardiovascular disease, obesity, diabetes, and colorectal cancer.

The current scientific literature consistently highlights the superiority of plant-based and Mediterranean dietary patterns over the Western diet in promoting gut health and preventing NCDs. However, the relationship between diet and gut microbiota is complex and influenced by numerous factors, including genetics, environment, and lifestyle. Future research should aim to deepen our understanding of these interactions and explore how targeted dietary interventions can be leveraged as therapeutic strategies to modulate gut microbiota composition and improve health outcomes. Moreover, identifying specific gut microbiota profiles associated with various health states could lead to the development of personalized nutrition approaches for the prevention and management of NCDs.

## Figures and Tables

**Table 1:** Effect of dietary types on gut microbiota composition.

Dietary Type	Sample	Study Groups	Main Findings	Reference
Plant-based	31 participants, with ischemic heart disease	16 individuals followed a vegetarian diet (94% men, median age of 67 years), 15 followed a meat diet (93% men, median age of 68 years)	↑ *Ruminococcaceae*, *Akkermansiaceae*, and *Lachnospiraceae* in individuals following a vegetarian diet	[91]
Plant-based	168 overweight participants	84 individuals followed a vegan diet (82% women, mean age of 52.9 years), 84 were in the control group (88% women, mean age of 57.5 years)	↑ *Faecalibacterium prausnitzii*, and ↓ *Bacteroides fragilis* in individuals following a vegan diet	[92]
Plant-based	27 pregnant women	9 women followed a vegetarian diet (mean age of 33 years), 18 were omnivorous (mean age of 34 years)	↑ *Roseburia* and *Lachnospiraceae* ↓ *Collinsella* and *Holdemania* in vegetarian women	[93]
Plant-based	288 participants	265 individuals followed a vegan diet and meditation (139 women, mean age of 40.9 years), 23 were in the control group (13 men, mean age of 42 years)	↑ *Bifidobacterium*, *Lactobacillus*, *Streptococcus*, *Collinsella*, and *Ruminococcaceae* in individuals following a vegan diet	[94]
Mediterranean	69 participants	23 obese individuals followed a hypocaloric MD (20 women, mean age of 53 years), 46 were normal-weight individuals (40 women, mean age of 49 years)	*↓ Firmicutes* ↑ *Bacteroidetes* and *Proteobacteria* in obese individuals following hypocaloric MD	[95]
Mediterranean	82 overweight and obese participants	43 individuals followed MD, 39 followed a regular diet (43 women, 39 men, mean age of 43 years)	↑ *Faecalibacterium prausnitzii*, *Lachnospiraceae*, and *Roseburia* in individuals following MD	[96]
Mediterranean	294 participants with dyslipidemia or abdominal obesity	96 individuals followed healthy dietary guidelines (88% men, mean age of 51.34 years), 95 MD (87.5% men, mean age of 51.74 years), 95 Green MD (89.5% men, mean age 50.68 years)	↑ *Prevotella* and ↓ *Bifidobacterium* in individuals following MD and Green MD	[97]
Mediterranean	60 participants with rheumatoid arthritis	20 individuals adhered highly to an MD (100% women, mean age 66 years), 40 had low or moderate adherence to an MD (75% women, mean age of 59.5 years)	↓ *Lactobacillaceae* and *Prevotella copri* in individuals with higher adherence to an MD	[98]
Western	307 healthy participants	Individuals were divided into 3 groups based on fat intake, a 1:1:1 ratio (age between 18 and 35 years, 52% were women)	↓ *Faecalibacterium*, ↑ *Bacteroides* and *Alistipes* in individuals following a high-fat diet	[99]
Western	81 participants	40 individuals consumed refined grains (25 men, mean age of 54 years), 41 consumed whole grains (24 men, mean age of 55 years)	↑ *Lachnospira* and ↓ *Enterobacteriaceae* in individuals consuming whole grains instead of refined grains	[100]
Western	117 overweight participants	59 individuals ate fried meat 4 times/week (55.9% women, mean age of 21.1 years) 58 had limited intake of fried meat (53.5% women, mean age of 21.7 years)	↑ *Veillonella*, *Dorea*, and *Dialister* ↓ *Flavonifractor* and *Lachnospiraceae* in individuals, who consumed fried meat	[101]
Western	1371 participants	1371 individuals consumed added sugars (577 with urinary sugar biomarker), 1086 sugar-sweetened beverages (SSBs), 1085 artificially sweetened beverages (ASBs), aged between 18 and 70 years	↓ *Lachnobacterium*, increased *Firmicutes/Bacteroidetes* ratio in individuals consuming sugar-sweetened beverages	[102]

Abbreviations and explanations: MD—Mediterranean diet, ↑—higher abundance, ↓—lower abundance.

**Table 2:** *Firmicutes*/*Bacteroidetes* ratio, and SCFA and TMAO production change with different dietary types.

	Plant-Based Diet	Mediterranean Diet	Western Diet
*Firmicutes*/*Bacteroidetes* ratio	↓ [103]	↓ [104]	↑ [105]
SCFA production	↑ [89]	↑ [106]	↓ [107]
TMAO production	↓ [90]	↓ [108]	↑ [109]

Abbreviations and explanations: SCFAs—short-chain fatty acids, TMAO—trimethylamine-N-oxide, ↑—increase, ↓—decrease.

## Data Availability

No new data were created.
